# Contamination factors associated with surviving bacteria in Thai commercial raw pet foods

**DOI:** 10.14202/vetworld.2020.1988-1991

**Published:** 2020-09-25

**Authors:** Suppada Kananub, Nayika Pinniam, Sitthiporn Phothitheerabut, Praphaphan Krajanglikit

**Affiliations:** 1 Department of Veterinary Public Health, Faculty of Veterinary Medicine, Kasetsart University, Kamphaeng, Nakhon Pathom, Thailand; 2 Kamphaeng Veterinary Diagnostic Center, Faculty of Veterinary Medicine, Kasetsart University, Kamphaeng, Nakhon Pathom, Thailand

**Keywords:** foodborne pathogen, freeze-dried product frozen product, meat types, raw pet food

## Abstract

**Aim:**

This study aimed to identify the surviving bacteria in commercial raw pet food and to analyze the factors associated with their contamination.

**Materials and Methods:**

A total of 17 samples from 12 brands available in Thailand were randomly selected for analysis. Fifteen samples were frozen products and two were freeze-dried. The total bacterial counts (TBCs) of *Clostridium* *perfringens, Campylobacter* spp., *Staphylococcus aureus*, *Escherichia coli, Salmonella* spp., *Listeria* spp., and *Listeria* *monocytogenes* were measured. Association between the bacterial profile and feed ingredients, as well as with product types, was analyzed by Chi-squared and Fisher’s exact tests.

**Results:**

*Campylobacter* was not found in any product, whereas *Salmonella* spp. and *Listeria* spp. showed the highest prevalence with respect to the standard’s limits. The TBC was significantly related to the type of the products (frozen or freeze-dried), and *S. aureus* and *L. monocytogenes* were significantly related to a chicken-based diet.

**Conclusion:**

Pet food contamination can occur during the manufacturing process, storage, or even preparation. The freezing and drying processes may reduce, but not eradicate, the bacterial contamination in raw pet food. These results emphasize the need for quality control in the manufacturing process and show the importance of personal hygiene for the pet owner to reduce health risks.

## Introduction

Raw pet food is commonly used to feed pet animals and can be variably named as bone and raw feed, biologically appropriate diets, or raw meat-based diets [1-3]. Pet owners can prepare raw meat food themselves or can purchase ready-to-feed commercial products. The advantages of raw food include better palatability, digestibility, health benefits, and its natural origin; however, there is no substantial evidence to prove these claims [2,4,5]. Freezing is a process that reduces the product temperature to below 0°C, leading to the water inside the raw meat to form ice. It is a widely accepted process for the preservation and inactivation of pathogens, including bacteria, yeast, and parasites, known to contaminate food products [6-8]. Although the number of pathogens is reduced through the freezing process, certain pathogens can remain dormant for a long time while maintaining their pathogenicity [8,9], multiplying once the product is removed from storage and thawed. This represents a real risk of pathogen transfer from the food products to the animals or their owners, which could be associated with potential health problems [10-12].

*Escherichia coli*, *Salmonella* spp., *Clostridium* spp., *Campylobacter* spp., and *Listeria* spp. are foodborne pathogens, which can be found in raw meat diets, that can result in significant health risks [2,4,12]. In 2017, in the United Kingdom, an incidence of pathogen contamination was reported to affect 80% of the raw meat products, which was five-fold higher than the incidence of such contaminants in complete feed [13]. Moreover, a recent study reported the incidence of tuberculosis in cats due to *Mycobacterium bovis* contaminated raw meat diet [14]. These observations reaffirm the concerns about using raw meat food for pets. Indeed, Freeman *et al*. [2] have found that contamination of raw pet food is directly associated with the presence of raw meat as an ingredient. For example, *Salmonella* spp. contaminated up to 40% of raw chicken meat products, whereas it contaminated <5% of pig or beef products.

Taking all these findings into consideration, this study aimed to identify the bacteria present in 12 of the 25 commercial raw pet food brands available in Thailand and to analyze the factors associated with such contamination.

## Materials and Methods

### Ethical approval

Ethical approval was not required for this study.

### Study period and location

The samples were collected from areas of Bangkok, Nontaburi and Ratchaburi province in the central part of Thailand. The samples were analyzed at Kamphaeng Saen Veterinary Diagnostic Center. All processes were performed during October 2019 to February 2020.

### Sample collection

The sample size was defined by the equation of disease detection that is used for identifying the incidence of an event. This calculation was performed byProMESA software (Instituto Nacional de Tecnología Agropecuaria; Argentina and EpiCentre, IVABS, Massey University; New Zealand), based on the minimum expected prevalence of 0.1 bacterial contamination in raw meat food and the total of 25 brands of pet food available in Thailand at p<0.05. A total of 17 samples of 12 different brands, which comprised 15 frozen and two freeze-dried products, were randomly selected and bought from an online shop and physical stores. Nine samples comprised one type of meat, seven samples were composed of two types of meat, and nine samples comprised three types of meat. About 40% of the samples were composed of chicken, 10% were duck meat, and 50% were fish, whereas beef accounted for approximately 25% of the samples (Table-1). All products were contained in a vacuum package, except for the freeze-dried products. The frozen products were kept at −20°C, and the freeze-dried were refrigerated at 4°C until further analysis.

**Table-1:** Number of samples classified by the ingredient.

Types of food	Types of meat	Number of samples
Frozen food	Chicken	2
	Fish	5
	Chicken and fish	2
	Chicken and beef	1
	Fish and beef	3
	Fish and duck	1
	Chicken, fish, and duck	1
Freeze-dried food	Chicken	1
	Fish	1

### Sample analysis

Frozen samples were thawed in a refrigerator at 4°C for 24 h, and the freeze-dried products were rehydrated following product instructions using sterile water at 25°C. The samples were aseptically subdivided according to analytical techniques.

Each sample was analyzed for eight profiles: Total bacterial count (TBC), *Clostridium* *perfringens, Campylobacter* spp., *Staphylococcus aureus*, *E. coli, Salmonella* spp., *Listeria* spp., and *Listeria* *monocytogenes*. Quantification of TBC (Association of Official Analytical Chemists [AOAC] official method SM–N° 2008.10), *S. aureus* (AOAC method 975.55), and *E. coli* (AOAC method M–N° 2009.02) was conducted using an automated TEMPO instrument (bioMérieux, Marcy-l’Étoile, France). The detection limit of the analysis of *S. aureus* and *E. coli* was 100 CFU/g. The identification of *Campylobacter* spp. was performed using the conventional method (ISO10272:1995-1). Qualitative analysis of *Salmonella* spp., *Listeria* spp., and *L. monocytogenes* (AOAC method 2013.10) was performed using a VIDAS system (bioMérieux) based on the enzyme-linked fluorescent assay. In addition, the conventional method (AOAC method 976.30) was used to identify *C*. *perfringens*.

### Statistical analysis

The microbiological profiles were analyzed using descriptive statistics. Food quality was evaluated using the microbiological criteria quality standards for foods and food containers [15]. The qualitative criteria of *Campylobacter* spp., *Salmonella* spp., *Listeria* spp., and *L. monocytogenes* were set at non-detectable bacteria in 25 g of raw food. The quantitative criteria for TBC and *C. perfringens* were accepted when the counts were <5×10^6^ and 1000 CFU/g of raw food, respectively, whereas for *E. coli* and *S. aureus* values lower than 100 CFU/g raw food were accepted [15].

For the analysis of the factors associated with the bacterial profile, the Chi-squared test and Fisher’s exact test were used; the relationship was deemed significant at p<0.05. Statistical analyses were conducted using the STATA software version 11.0 (StataCorp, College Station, TX, USA).

## Results

Table-2 presents the number of raw pet foods considered using the criteria of microbiological quality standards for foods and food containers. Overall, 80% of the samples had a higher TBC than the standard criteria, and the data were left-skewed. The median was 181×10^6^ CFU/g, while Q1 and Q3 were 80×10^6^ and 239×10^6^ CFU/g, respectively. About 40% of the samples showed over 100 CFU/g for *E. coli* and *S. aureus*, with the highest contaminations by *E. coli* and *S. aureus* were 7.4×10^4^ and 0.26×10^4^ CFU/g, respectively. *E. coli* and *S. aureus* contaminations were not further detailed due to high uncertainty on the obtained data. *C. perfringens* was detected in only two samples at 100 and 150 CFU/g that passed the standard criteria, while all samples were negative for *Campylobacter*. However, about half of the samples had detectable levels of *Salmonella* spp. and *Listeria* spp., and 20% were positive for *L. monocytogenes*.

**Table-2:** Number of raw meat food exceeded the microbiological quality standard for foods and food containers.

Pathogens	Number of samples (n=17)
Total bacterial count^†^	14
*Escherichia coli*^‡^	7
*Staphylococcus aureus*^‡^	7
*Clostridium perfringens*^§^	0
*Campylobacter* spp.^¶^	0
*Salmonella* spp.^¶^	9
*Listeria* spp.^¶^	9
*Listeria monocytogenes*^¶^	3

† The standard considered at <5.0×10^6^ CFU/g,

‡ The standard considered at <100 CFU/g,

§ The standard considered at <1000 CFU/g,

¶ The standard considered as undetected in 25 g

The analyses revealed that only TBC was significantly associated with the types of food that was frozen or freeze-dried (p<0.05). Overall, 90% of the frozen products had greater TBC than the allowed limit, but TBC was below the standard criterion in all freeze-dried products. Furthermore, *S. aureus* and *L. monocytogenes* were significantly related to the chicken-based diet (p<0.05), and chicken-related feed was highly contaminated with *S. aureus*. Contamination by *L*. *monocytogenes* was identified in approximately 43% of the products with chicken. In contrast, all products with other meats were free from this pathogen.

## Discussion

All pathogens, except the *Campylobacter* species, were detected in the 12 samples tested of commercial raw pet food. *Listeria* spp. and *Salmonella* spp. were the two bacteria most commonly found in the samples at higher amounts than the allowed standard limits. According to the previous reports, the pathogens most commonly found in raw meat food for dogs and cats are *E. coli*, *Listeria* spp., *L. monocytogenes*, *S. aureus*, *C. perfringens, Salmonella* spp., *Bacillus* spp., *Klebsiella* spp., *Salmonella* spp., *Flavobacterium* spp., *Campylobacter* spp., and *Pseudomonas* spp. [7,10,12,16]. Furthermore, high bacterial prevalence was reported for Enterobacteriaceae, *Listeria* spp., *L. monocytogenes*, and *Enterococcus faecalis* regardless of the type of raw meat [10,17]. A lack of hygiene and sanitation in manufacturing units could be responsible for causing this high prevalence of the different contaminants in raw meat products [18]. In addition, differences in the contamination levels could be the consequence of the study areas, types of meat, or the scope of the study.

In this study, the TBC was significantly associated with the origin of the products and whether they were frozen or freeze-dried. The survival of pathogens in the freeze-dried products was lower than in frozen products, which may result from the dried meat environment not being suitable for microbial growth due to its low moisture content. In addition, other bacterial profiles were not significantly related to the types of products tested. These findings contrast with Freeman *et al*. [2], who did not encounter differences in the bacterial levels between freeze-dried, frozen, and raw meat products. Nevertheless, the maximum reduction in microbial growth has been observed during the complete drying process [19]. Gram-negative bacteria are more sensitive to dehydration because of their cell wall structure [9,20]. Although sanitation is an essential factor for the manufacturing process in the food industry [8,21,22], the production processes for raw pet food may not inactivate the bacteria completely.

Regarding the ingredients comprised in the pet foods, raw chicken showed a significant relationship with *S. aureus* and *L. monocytogenes* contamination (p<0.05). Other studies have reported *E. faecalis* and coliforms as the main organisms in a chicken-based diet [7,10]. Moreover, in another study, the principal pathogenic bacteria in chicken meat were *Salmonella* spp. and *Campylobacter* spp. [16]. In this study, we considered only bacteria for the microbiological standards of food and food containers in Thailand, which might explain the different results. Furthermore, the type of contamination depends on where the contamination occurs in the production line and on the bacterial strain [10,18,19,22].

Duck and beef meat might result in less contamination by any organism. The previous studies revealed that the most prevalent bacteria in duck and beef products were *Aeromonas* spp., *Pseudomonas* spp., *Leuconostoc mesenteroides*, and *E. coli* [10,23]. In fish, the most common contaminants were *S. aureus*, *Bacillus* spp., *Salmonella* spp., *Enterobacter* spp., and Flavobacterium, regardless of the microbial standard limit [7]. The bacterial contaminants in raw meat were also detected in animal feces [4,24]. The shedding of the bacteria from the dogs fed on raw food was approximately 6 times more likely than dogs not fed with raw food [25]. The bacteria not only cause diseases in pets, but the owners in contact with their pet or leftover food can also be affected, in particular children, the elderly, and immunocompromised people [4,16,24]. In addition, *L. monocytogenes* was suspected to be the predisposing cause of osteomyelitis in dogs, whereas gastrointestinal problems existed in *Salmonella-*infected cats that consumed commercial raw meat [5,26].

Raw meat also carries antimicrobial resistance strains and parasites that severely impact humans [16,17]. Raw pet food has been mentioned as being beneficial for animal health; therefore, it is widely popular [2]. However, its contamination during production and the potential of bacterial growth during storage and preparation are critical issues [10,11,21]. Nevertheless, commercial pet food is recommended over homemade food [2]. In this study, the small number of samples may have contributed for the reduced statistical power of the analyses, resulting in non-significant associations in a few samples. Moreover, only two brands of freeze-dried diets were available for analysis.

## Conclusion

This study identified the bacteria responsible for the contamination of commercial raw pet foods that can pose a risk to animal and human health. Almost all systems can be aggravated according to the contamination of many kinds of foodborne bacteria. Not only pet animals can be infected, but the humans who contact with the contaminated product are also at risk. Importantly, the bacterial contamination of pet foods can occur at any step, from the manufacturing process to its handling. Therefore, this study highlights the importance of quality control in food production and personal hygiene of pet owners, during the preparation and handling of pet foods, to reduce potential health risks for the animals and their owners.

## Authors’ Contributions

SK planned and administered the entire research work. NP, SP, and PK carried out the laboratory work and sample collection. All authors have read and approved the final manuscript.
